# Fluorimetric Method for the Determination of Histidine in Random Human Urine Based on Zone Fluidics

**DOI:** 10.3390/molecules25071665

**Published:** 2020-04-04

**Authors:** Antonios Alevridis, Apostolia Tsiasioti, Constantinos K. Zacharis, Paraskevas D. Tzanavaras

**Affiliations:** 1 Laboratory of Analytical Chemistry, School of Chemistry, Faculty of Sciences, Aristotle University of Thessaloniki, GR-54124 Thessaloniki, Greece; alevridi@chem.auth.gr (A.A.); atsiasioti@gmail.com (A.T.); 2 Laboratory of Pharmaceutical Analysis, Department of Pharmaceutical Technology, School of Pharmacy, Aristotle University of Thessaloniki, GR-54124 Thessaloniki, Greece; czacharis@pharm.auth.gr

**Keywords:** histidine, random human urine, zone fluidics, *o*-phthalaldehyde, derivatization, stopped-flow, fluorimetry

## Abstract

In the present study, the determination of histidine (HIS) by an on-line flow method based on the concept of zone fluidics is reported. HIS reacts fast with *o*-phthalaldehyde at a mildly basic medium (pH 7.5) and in the absence of additional nucleophilic compounds to yield a highly fluorescent derivative (*λ*_ex_/*λ*_em_ = 360/440 nm). The flow procedure was optimized and validated, paying special attention to its selectivity and sensitivity. The LOD was 31 nmol·L^−1^, while the within-day and day-to-day precisions were better than 1.0% and 5.0%, respectively (*n* = 6). Random urine samples from adult volunteers (*n* = 7) were successfully analyzed without matrix effect (<1%). Endogenous HIS content ranged between 116 and 1527 μmol·L^−1^ with percentage recoveries in the range of 87.6%–95.4%.

## 1. Introduction

l-Histidine (HIS) is an essential amino-acid that has proven to play unique roles in human organism [1,2]. Representative examples include: (i) when it is co-administered with iron, it helps towards the most rapid increase in its plasma levels in patients with anemia, especially to those with chronic kidney failure [3]; (ii) in vitro studies have demonstrated that due to its metal chelating properties HIS is the most effective hydroxyl radical scavenger compared to several amino acids that were examined [4]; (iii) it has significant anti-inflammatory properties, mainly due to the production on histamine through enzymatic decarboxylation [5]; and (iv) a recent study also revealed impressive improvement of the metabolic syndrome through supplementation with HIS [6,7]. The critical role of HIS along with arginine and tryptophan in the strengthening of the immune system particularly during cancer immune-therapies has very recently been reviewed [8]. Detection of histidine in urine samples is associated with the diagnosis of histidine metabolism disorders, particularly ‘histidinemia’ at elevated levels in physiological fluids (normal level: 130–2100 mM in urine) [9] or with the level of histamine secretion in allergic patients [10].

From an analytical chemistry point of view, a search through the scientific literature revealed a continuous interest in the development of novel methods for the determination of HIS. Many of the recently reported methods are indirect based on the well-known affinity and interaction of HIS with metal ions such as Cu(II) and Ni(II). For example, a colorimetric naked-eye chemosensor was based on a thiazolylazo dye–Ni(II)–HIS system visual color change from red to yellow. Although amino acids were reported not to interfere, no validation and application to real samples was included [11]. In a similar manner, HIS was found to reverse the quenching effect of Cu(II) ions on the fluorescence of semiconductor quantum dots (QD), enabling the analysis of HIS at the micromolar level [12]. According to the authors, although the optimal pH of the sensor favors the selectivity against –SH containing amino acids (e.g., cysteine), the endogenous Fe and Cu content of biological samples should always be taken into account for living cell imaging applications.

Automation of analytical methods through flow-based configurations is always attractive and up-to-date due to the unique advantages of high throughput, strict and precise control of experimental conditions, enhanced selectivity based on kinetic differentiations, etc. Among flow-based methods the concept of Zone Fluidics (ZF) offers some additional features including effective manipulation of reactants zones at the micro-liter level, adaptation of various chemistries through single channel configurations, minimization of waste generation, even efficient coupling to separation techniques as a front-end sample preparation platform [13,14]. ZF coupled to analytical derivatization have proven as an advantageous alternative to the development of robust and reliable methods for a variety of analytes such as hydrazine [15], adamantane derivatives [16], dopamine [17], creatinine [18], etc.

The goal of the present study was to develop, validate, and apply a reliable and fast method for the determination of HIS in random urine samples. To achieve this task, we have chosen to automate the reaction between HIS and *o*-phthalaldehyde (OPA) through a ZF flow platform. OPA has been reported to react with HIS at a basic pH to form a relatively stable and highly fluorescent derivative [19]. The above-mentioned reaction is promising since it proceeds without the need of nucleophilic compounds and is therefore selective against numerous primary amino-group containing analytes that react with OPA through the traditional mechanism [20]. Additionally, histamine that is structurally analogous form an unstable derivative at alkaline medium requiring acidification prior to detection [21], while glutathione reacts with OPA at a significantly more basic pH [22].

From a literature point of view, to the best of our knowledge, only one method for the determination of HIS has been published based on ZF [23]. It is based on chemiluminescence (CL) detection of a rather complicated chemical system—HIS increases the catalytic activity of Mn(II) salts in the CL reaction with luminol-hydrogen peroxide in the presence of dioximes in sodium borate medium. Although the authors report application in real beer samples, the selectivity of the method should be of concern in real world applications since—as could be expected—some common metal ions that are well known to act catalytically in luminol-based reactions (Fe(III), Cu(II), Co(II), etc.) and amino acids interfere at comparable of even lower levels to HIS.

## 2. Results and Discussion

### 2.1. Preliminary Experiments

Preliminary experiments were carried out in the batch mode to verify the reaction of OPA and HIS in the absence of nucleophilic compounds. A representative off-line fluorimetric detect (FL) spectrum is shown in Figure S1 (Supplementary Material). All subsequent experiments were performed at λ_ex_ = 360 nm and λ_em_ = 440 nm.

In a second series of preliminary studies, it was confirmed that the reaction can proceed under zone fluidic conditions and that was promising for further development and optimization. Initial ZF experiments were performed under the following starting variables: [OPA] = 10 mmol·L^−1^, pH = 7–9, T = ambient temperature, V = 50 μL for all zones, and [HIS] = 10 μmol·L^−1^. The order of mixing proved to have negligible impact on the sensitivity and the aspiration order of OPA/Buffer/Sample was adopted for all subsequent experiments.

### 2.2. Development of the ZF Method

The development of the ZF method involved experimental studies of the various chemical and instrumental parameters that are expected to affect the performance of the procedure, such as: the pH of the reaction (6.0–9.0), the reaction time (0–120 s), the reaction temperature (25–40 °C), the amount concentration of OPA (5–15 mmol·L^−1^), and the volumes of the sample (50–125 μL) and the OPA/Buffer (25–75 μL).

**Figure 1:**
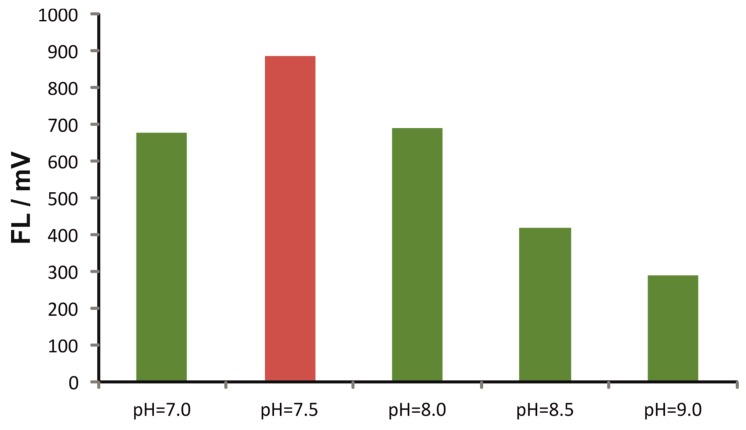
Effect of the pH on the fluorescence intensity of histidine–*o*-phthalaldehyde (HIS–OPA) derivative under flow conditions.

As can be seen in Figure 1, the reaction is clearly favored at a mildly basic pH of 7.5. It should be noted that our findings under flow conditions are not in absolute agreement with the batch method, since Hakanson et al. have reported an optimum reaction pH of 11.2–11.5 [19]. Such variations are common in flow methods due to the kinetic character of the procedures, compared to the “thermodynamic” batch analogues. For example, the reaction can be favored kinetically at a mild alkaline pH, while the more alkaline pH reported in [17] might provide a more stable product at a lower reaction rate. Additionally, this is an important feature in terms of selectivity since most of the OPA reactions take place in significantly more alkaline pH regions. For example, Glutathione (GSH) reacts with OPA at pH values > 8.0 and the highest sensitivity is achieved at pH > 12.0.

**Figure 2:**
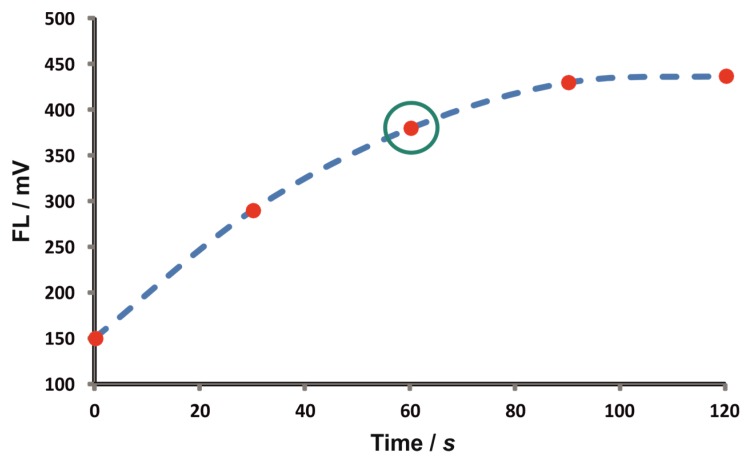
Effect of the reaction time under stopped-flow conditions; *t* = 0 corresponds to the delivery of the reaction product to the detector through the reaction coil without the stopped-flow step.

The effect of the reaction time was investigated under stopped-flow conditions. In brief, following aspiration of the zones in the holding coil (HC) of the ZF setup, the flow was reversed for 30 s at 0.6 mL·min^−1^. The reaction mixture is “trapped” in the reaction coil (RC) and the flow is stopped for increasing time intervals. As can be seen in Figure 2, there is a non-linear increase in the range of 0–90 s, while the signals practically leveled-off thereafter. A stopped-flow reaction time of 60 s was selected as a compromise between sensitivity and sampling throughput. On the other hand, variation of the reaction temperature up to 60 °C offered a ca. 25% sensitivity enhancement and the latter value was adopted by thermostating the reaction coil using an HPLC column oven (see experimental section).

The volumes of the samples and reagents zones are an important variable that affects both the mixing efficiency and the dispersion/sensitivity. The sample injection volume vs. the fluorescence intensity had an almost linear profile in the range of 50–125 μL (at 2 μmol·L^−1^ HIS) and the signals practically doubled in this range. A sample injection volume of 100 μL was selected in terms of satisfactory sensitivity, sampling rate, and sample consumption. On the other hand, the volumes of the OPA reagent and the buffer had a rather moderate effect on the method. In both cases, 50 μL were selected taking into account the sensitivity and the consumption of the reagents.

Finally, the amount concentration of the reagent was investigated in the range of 5–15 mmol·L^−1^. The criteria were the sufficient excess of the reagent and its effect on the kinetics of the reaction (sensitivity). The experimental results confirmed that the method was unaffected at OPA amount concentrations >10 mmol·L^−1^, where the signals reached a plateau. The latter value was therefore selected for subsequent validation experiments.

An overview of the studied instrumental and chemical variables, the investigated range and the selected values is included in Table 1.

### 2.3. Validation of the ZF Method

The proposed method has been validated for linearity, limits of detection (LOD) and quantification (LOQ), precision, selectivity, matrix effect, and accuracy.

#### 2.3.1. Linearity, LOD and LOQ

The method proved to offer satisfactory linearity in the range of 125–2000 nmol·L^−1^ (20–310 μg·L^−1^) HIS. The regression equation was obtained in a “cumulative” way by incorporating the results from more than 90 standard solutions analyzed in different working days (*n* = 8). In this way, the calibration curve is more representative including potential day-to-day variations. The following regression Equation (1) was obtained:

*F* = 420.1 (±3.4) [HIS] + 63.1 (±3.9), *r*^2^ = 0.994 (1)

where *F* is the fluorescence intensity as measured by the detector. Linearity was further validated using the back-calculated concentrations (residuals). The percent residuals were distributed randomly around the “zero axis” and ranged between −13.2% and +9.5%. It should be noted that the highest positive and negative values correspond to residuals at the lowest point of the calibration curve close to the LOQ of the method.

The LOD and LOQ was estimated based on the following Equations (2):

LOD = 3.3 × SD_b_/*s* and LOQ = 10 × SD_b_/*s* (2)

where SD_b_ is the standard deviation of the intercept and *s* is the slope of the respective regression lines. The calculated LOD/LOQ for the analyte was 31 and 93 nmol·L^−1^, respectively, corresponding to 4.8 and 14.4 μg·L^−1^ HIS.

#### 2.3.2. Within and between Day Precisions

The within-day precision was validated at the 1.0 μmol·L^−1^ level by repetitive injections (*n* = 8). The relative standard deviation (RSD) was 0.5 % (see Figure S2 in supplementary section).

The day-to-day precision was evaluated by independent calibration curves obtained at different working days (*n* = 8). As can be seen in Table S1 (supplementary section), the RSD of the regression slopes was 4.2%, verifying the repeatability of the flow procedure.

#### 2.3.3. Selectivity and Matrix Effect

The selectivity/matrix effect of the proposed method was evaluated towards three axes:

(i) against amino-acids and biogenic amines that can potentially react with OPA;
(ii) using an artificial urine matrix spiked with the analyte;
(iii) using a pooled human urine sample also spiked with the analyte.

As can be seen in Table 2, several amino-acids and biogenic amines were examined on the basis of their reaction with OPA at the optimal values set for the determination of HIS. All compounds were set at 10.0 μmol·L^−1^ (10-fold excess compared to HIS) with the exception of Histamine and Glutathione that were examined at equimolar concentrations to HIS (1.0 μmol·L^−1^). Based on the experimental results the selectivity factor (S.F.) for each potential interfering compound (INT) was calculated according to the following Equation (3):

(3) $$S.F.=\frac{FL\left(\mathrm{HIS}\right)}{FL\left(\mathrm{INT}\right)}\times \frac{c\left(INT\right)}{c\left(HIS\right)}\;\;$$

The results can be categorized as follows:

(i) Alanine, Lysine, Threonine, Serine, and Tyrosine do not seem to react with OPA at the experimental conditions of the ZF method since the obtained signals were not different compared to the blank values.
(ii) Glycine, Cysteine, and Glutamate react with OPA, but the selectivity factors are high, ranging between 32–148.
(iii) Histamine and Glutathione seem to cause the most serious interference at equimolar levels to HIS. Both compounds are known to react with the tagging reagent in the absence of nucleophilic compounds, yielding fluorescent derivatives [22,24]. However, the selectivity factors of ca. four are quite satisfactory taking into account the fact that HIS is in great excess in the real samples compared to the above compounds. The derivative of Histamine is more stable and with higher fluorescence at acidic medium, while the reaction of Glutathione is favored kinetically in more alkaline pH.

To evaluate the matrix effect, a widely accepted artificial urine sample has been prepared as described in the experimental section. As can be seen in Table 3, the matrix effect was examined at several dilution factors of the artificial urine at HIS concentrations covering the linearity range of the method. The slopes of the matrix matched curves were compared against the aqueous regression line and the matrix effect was evaluated as the relative error (%). The experimental results confirmed significant negative matrix effect (signal suppression) at 1:5 and 1:10 dilutions of the artificial urine ranging between −35% and −55%. On the other hand, the matrix effect was minimized (<5%) for dilution factors higher than 1:100. The obtained results were quite promising since the sensitivity of the method is high and real urine dilutions of >500-fold are expected to be necessary for quantification of HIS based on its reported levels in the literature [25].

Further evaluation of the matrix effect was carried out using a real pooled urine sample (*n* = 8). In brief, 500 μL of each urine sub-sample were transferred in a centrifuge tube and mixed. An equal volume of ice-cold acetonitrile (4 mL) was added to precipitate proteins followed by centrifugation. The obtained solution was diluted 250-fold and spiked with HIS in the range of 250–1500 nmol·L^−1^. The experimental regression Equation (4) was:

*F* = 417.5 (±3.1) [HIS] + 242.8 (±2.9), *r*^2^ = 0.999 (4)

offering a <1% matrix effect compared to the cumulative aqueous calibration curve and enabling its practical use for analyzing the individual real urine samples.

#### 2.3.4. Accuracy of the ZF Method

The accuracy of the method was validated at three concentration levels (500, 1000, and 1500 nmol·L^−1^) preparing two independent series of samples in the pooled urine matrix. Quantification was carried out using the cumulative aqueous calibration curve described in Section 2.3.1. The experimental results are presented in Table 4 and are quite satisfactory for this type of analysis with percent recoveries ranging between 87.6% and 95.4%.

### 2.4. Applications of the ZF Method

Seven random urine samples from male and female volunteers were analyzed according to the optimized and validated method described above. Samples were preliminarily screened to determine the necessary dilution factor in order to fall within the range of the calibration curve. In all cases, the dilution factors were either 500 or 1000 being consistent with the validation of the method in terms of potential matrix effects. The experimental findings are tabulated in Table 5. The concentration of HIS ranged between 115.8 and 1527 μmol·L^−1^ and are in accordance with reference values for adults (>18 years) obtained in an extensive study (>800 samples) that has been reported in the literature [25].

## 3. Materials and Methods

### 3.1. Instrumentation

The ZF setup was consisted of the following parts: a Minipuls3 peristaltic pump (Gilson, Middleton, WI, USA); a low-pressure micro-electrically actuated 10-port valve (Valco, Brockville, ON, Canada); a RF-551 flow-through spectrofluorimetric detector operated at high sensitivity (Shimadzu, Kyoto, Japan); PTFE tubing was used for the connections of the flow configuration (0.5 or 0.7 mm i.d.); and Tygon tubing was used in the peristaltic pump. An HPLC column heater (Jones Chromatography, Hengoed, Mid Glamorgan, UK) was employed for the temperature control (60 ± 0.5 °C) of the reaction coil (100 cm/0.5 mm i.d.); the latter was tightly wrapped around the stainless-steel body of an old HPLC column (4.6 mm i.d.).

Control of the ZF system was performed through a LabVIEW (National Instruments, Austin, TX, USA) based program developed in house; while data acquisition (peak heights) was carried out through the Clarity^®^ software (version 4.0.3, DataApex, Prague, Czech Republic). Off-line spectra were recorded using a RF-5301PC batch spectrofluorophotometer (Shimadzu, Kyoto, Japan).

### 3.2. Reagents and Solutions

Histidine (HIS, 99%) was purchased by Sigma (St. Louis, MO, USA); *o*-phthalaldehyde (OPA, Fluka, Munich, Germany), KH_2_PO_4_ (Merck, Darmstadt, Germany), NaOH (Merck) and HCl (Sigma) were all of analytical grade. Doubly de-ionized water was produced by a Milli-Q system (Millipore, Bedford, MA, USA).

The standard stock solution of the analyte was prepared daily at the 1000 μmol·L^−1^ level in water. Working solutions were prepared by serial dilutions in water. The derivatizing reagent (OPA) was prepared at an amount concentration of 10 mmol·L^−1^ by firstly dissolving in 0.5 mL methanol and subsequently adding 9.5 mL water [22]. This solution was stable for a practical period of 3–4 working days at 4 °C in an amber glass vial. Phosphate buffer (100 mmol·L^−1^) was also prepared daily and regulated to the desired pH value (pH = 7.5) by drop-wise addition of NaOH (1 mol·L^−1^).

Synthetic urine (200 mL in water) was prepared according to the literature [26] and was consisted of the following (analytical grade): lactic acid (1.1 mmol·L^−1^), citric acid (2.0 mmol·L^−1^), sodium bicarbonate (25 mmol·L^−1^), urea (170 mmol·L^−1^), calcium chloride (2.5 mmol·L^−1^), sodium chloride (90 mmol·L^−1^), magnesium sulfate (2.0 mmol·L^−1^), sodium sulfate (10 mmol·L^−1^), potassium dihydrogen phosphate (7.0 mmol·L^−1^), di-potassium hydrogen phosphate (7.0 mmol·L^−1^), and ammonium chloride (25 mmol·L^−1^). The pH of the solution was adjusted to 6.0 by addition of 1.0 mol·L^−1^ HCl.

All other amino-acids and biogenic amines employed in the selectivity studies were of analytical grade and were supplied by Sigma. All solutions were prepared in water at the levels mentioned in the respective section.

### 3.3. ZF Procedure

**Figure 3:**
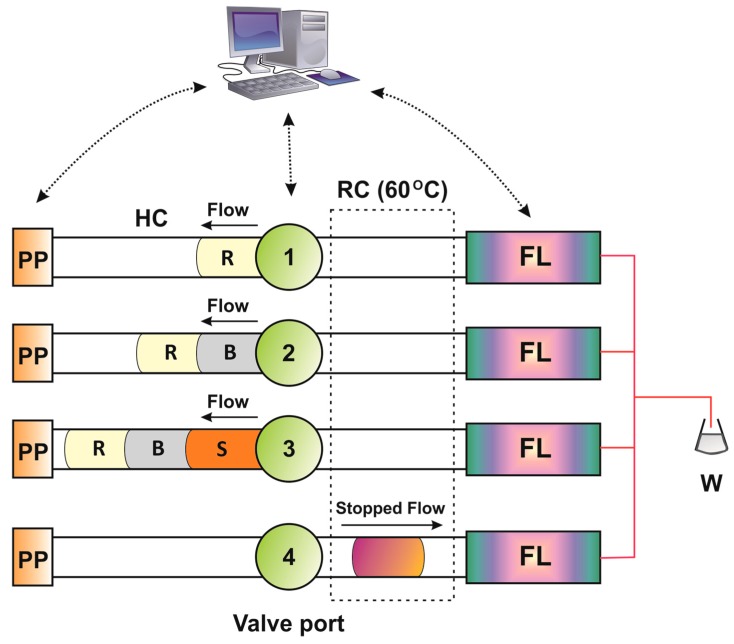
ZF sequence for the determination of HIS: PP = peristaltic pump; R = OPA reagent (10 mmol·L^−1^); B = buffer (100 mmol·L^−1^ phosphate, pH = 7.5); S = sample; HC = holding coil (300 cm/0.7 mm i.d.); RC = reaction coil (100 cm/0.5 mm i.d.); FL = fluorimetric detector (*λ*_ex_/*λ*_em_ = 360/440 nm); W = waste.

The experimental conditions of the optimized ZF sequence for the determination of HIS can found in Table 6 and are also depicted graphically in Figure 3. In brief, OPA (50 μL, 10 mmol·L^−1^), buffer (50 μL, 100 mmol·L^−1^ phosphate/pH = 7.5) and sample/standards (100 μL) were sequentially aspirated in the holding coil (HC).

Upon flow reversal, the stacked reaction zones were propelled towards the thermostated reaction coil (RC, 100 cm/60 °C) at a flow rate of 0.6 mL min^−1^ and the reaction was allowed to develop for 60 s under stopped-flow conditions. Downstream fluorimetric detection was carried out at λ_ex_/λ_em_ = 360/440 nm. The sampling throughput was 16 h^−1^.

### 3.4. Preparation of Urine Samples

Random urine samples were kindly donated voluntarily (no ethical approval was required) by male and female members of the laboratory. Samples where either processed immediately or stored at −20 °C until analysis [25].

Due to the selectivity and sensitivity of the developed method, sample preparation included the following simple and rapid steps:

(i) protein precipitation with addition of ice-cold acetonitrile (1 + 1);
(ii) centrifugation (4000 rpm, 10 min);
(iii) 500–1000-fold dilution depending on the levels of HIS in the real samples;
(iv) Analysis by the ZF method.

## 4. Conclusions

The developed on-line fluorimetric method for the determination of Histidine offers some interesting features:

1. It utilizes readily available reagents and due to the zone fluidics-based concept the consumption and generation of wastes is minimal compared to continuous flow techniques such as HPLC and Flow Injection Analysis.
2. The method is based on direct reaction and is advantageous compared to indirect methods based on inhibitory effects.
3. The high sensitivity of the method down to the nano-molar level enables the direct analysis of Histidine in human urine with minimum sample preparation.
4. The unique mechanism of the derivatization reaction excluded interference from most amino-acids and biogenic amines offering a highly selective platform for the determination of the analyte in the complicated samples without matrix effects.
5. Application in random urine samples was successful at a reasonable sampling frequency of 16 h^−1^.

## Figures and Tables

**Table 1:** Overview of the chemical and instrumental variables.

Variable	Studied Range	Selected Value
pH	7.0–9.0	7.5
Reaction time (stopped-flow, s)	0–120	60
Temperature (°C)	25–60	60
Sample volume (μL)	50–125	100
OPA volume (μL)	25–75	50
Buffer volume (μL)	25–75	50
OPA concentration (mmol·L^−1^)	5–15	10

**Table 2:** Selectivity of the proposed method.

Examined Compound	Amount Concentration (μmol·L^−1^)	FL (mV)	Selectivity Factor (S.F.)
Glycine	10	30	148
Glutamate	10	140	32
Alanine	10	NR ^a^	N/A
Lysine	10	NR	N/A
Threonine	10	NR	N/A
Cysteine	10	55	81
Serine	10	NR	N/A
Tyrosine	10	NR	N/A
Histamine	1	110	4.1
Glutathione	1	132	3.4

^a^ NR: not reacted.

**Table 3:** Matrix effect using artificial urine (250–2000 nmol·L^−1^).

	Dilution	Slope	Matrix Effect (%)
Aqueous curve	-	420.1	-
Artificial urine	1:5	185.3	−55.9
Artificial urine	1:10	271.8	−35.3
Artificial urine	1:100	441.3	+5.0
Artificial urine	1:250	428.5	+2.0

**Table 4:** Accuracy of the developed method.

Added (nmol·L^−1^)	Found (nmol·L^−1^)	Recovery (%)
500	438 (±21)	87.6
500	443 (±15)	88.6
1000	926 (±30)	92.6
1000	943 (±38)	94.3
1500	1431 (±51)	95.4
1500	1412 (±47)	94.1

**Table 5:** Analysis of random urine samples.

Sample	Histidine (μmol·L^−1^)	S.D. (*n* = 3)
Urine-A	384	12
Urine-B	1293	42
Urine-C	116	6
Urine-D	1527	50
Urine-E	629	18
Urine-F	445	21
Urine-G	873	34

**Table 6:** ZF steps for the determination of Histidine.

Time (s)	Pump Action	Flow Rate (mL min^−1^)	Volume (μL)	Valve Position	Action Description
0	Off	-	-	1	Selection of OPA reagent port
5	Aspirate	0.6	50	1	Aspiration of OPA in the holding coil
1	Off	-	-	2	Selection of buffer port
5	Aspirate	0.6	50	2	Aspiration of buffer in the holding coil
1	Off	-	-	3	Selection of sample port
10	Aspirate	0.6	100	3	Aspiration of sample in the holding coil
1	Off	-	-	4	Selection of detector port
30	Deliver	0.6	300	4	Propulsion of reaction mixture to reaction coil
60	Off	-	-	4	Stopped-flow step
120	Deliver	0.6	1200	4	Detection of derivative/end of measuring cycle

## Supplementary Materials

The following are available online, Figure S1: Representative FL off-line spectra of the HIS-OPA derivative. Figure S2: Within day precision at 1.0 μmol·L^−1^ HIS (*n* = 8). Table S1: Day-to-day precision of the developed method.
