# Supplementary material for: Fluorimetric Method for the Determination of Histidine in Random Human Urine Based on Zone Fluidics
Source: Molecules. 2020 Apr 4;25(7):1665. doi: 10.3390/molecules25071665 (PMC7180633; doi:10.3390/molecules25071665)
Supplement: Supplementary file 1 [file molecules-25-01665-s001.pdf]

molecules

Supporting Information

Fluorimetric Method for the Determination of Histidine in Random Human Urine Based on Zone Fluidics

Antonios Alevridis [1], Apostolia Tsiasioti [1], Constantinos K. Zacharis [2] and Paraskevas D. Tzanavaras [1,*]

1 Laboratory of Analytical Chemistry, School of Chemistry, Faculty of Sciences, Aristotle University of Thessaloniki, GR-54124 Thessaloniki, Greece; alevridi@chem.auth.gr (A.A.); atsiasioti@gmail.com (A.T.)
2 Laboratory of Pharmaceutical Analysis, Department of Pharmaceutical Technology, School of Pharmacy, Aristotle University of Thessaloniki, GR-54124 Thessaloniki, Greece; czacharis@pharm.auth.gr
* Correspondence: ptzanava@chem.auth.gr; Tel.: +30-2310997721, Fax: +30-2310997719

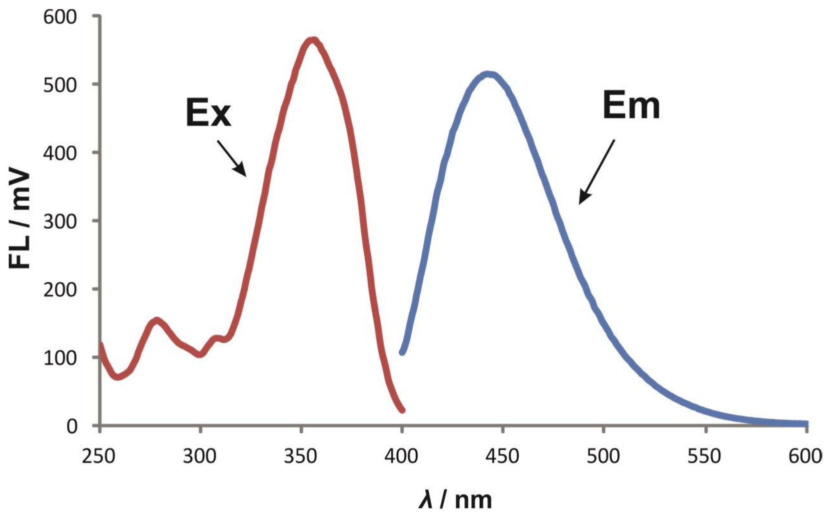

Figure S1. Representative FL off-line spectra of the HIS-OPA derivative.

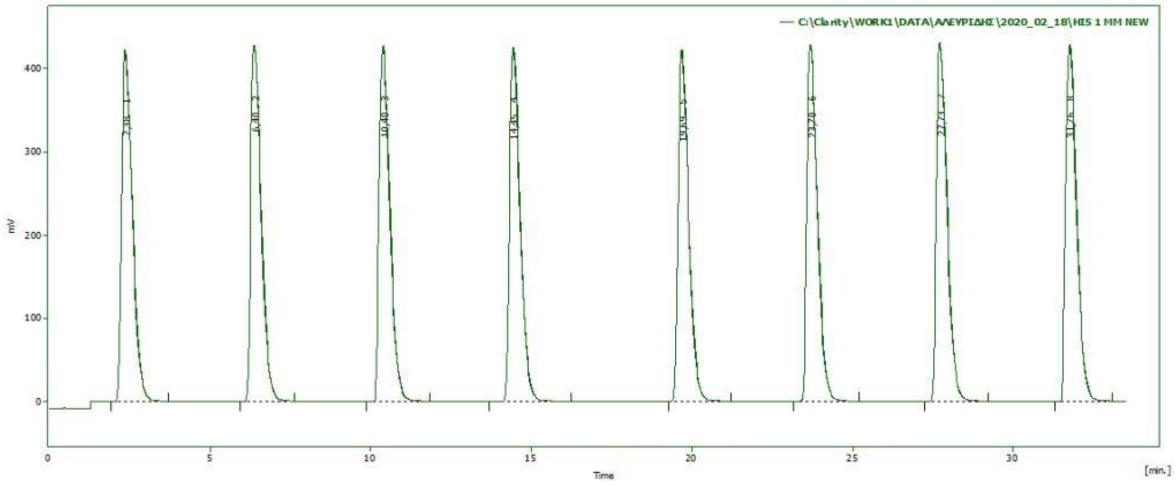

Figure S2. Within day precision at 1.0 μmol·L^{-1} HIS (n = 8).

Table S1. Day-to-day precision of the developed method

Working Day	HIS Slope
D1	444.6
D2	426.6
D3	423.0
D4	423.5
D5	423.1
D6	423.3
D7	425.3
D8	382.6
**Average (±SD)**	421.4 (±17.0)
**RSD (%)**	4.2
